# Using ICF to Describe Problems With Functioning in Everyday Life for Children Who Completed Treatment for Brain Tumor: An Analysis Based on Professionals' Documentation

**DOI:** 10.3389/fresc.2021.708265

**Published:** 2021-09-23

**Authors:** Ann-Christin Björklund, Mats Granlund, Sheila Judge Santacroce, Karin Enskär, Stefan Carlstein, Maria Björk

**Affiliations:** ^1^CHILD Research Group, Swedish Institute for Disability Research (SIDR), School of Health and Welfare, Jönköping University, Jönköping, Sweden; ^2^Department of Pediatric Hematology and Oncology, Uppsala University Hospital, Uppsala, Sweden; ^3^School of Nursing and Lineberger Comprehensive Cancer Center, The University of North Carolina at Chapel Hill, Chapel Hill, NC, United States; ^4^Department of Care Science, Faculty of Health and Society, Malmö University, Malmo, Sweden

**Keywords:** child, brain tumor, ICF, documentation, problem, everyday life

## Abstract

**Background:** Children treated for brain tumors often experience persistent problems affecting their activity performance and participation in everyday life, especially in school. Linking these problems to the International Classification of Functioning, Disability and Health (ICF) classification system can be described as affecting body function, activity performance, and/or participation. Services involved in the everyday life of the child have different focus and goals when meeting the child in context, which advantage the use of ICF to overcome this impediment to follow-up and provide comprehensive support for children who have completed treatment for a brain tumor.

**Aim:** The aim of the study was to use the ICF classification system to describe how professionals in healthcare, habilitation, and school document problems with everyday life functioning at body, activity, and participation levels for children who completed treatment for a brain tumor.

**Materials and Methods:** A retrospective review of records from healthcare, habilitation, and school concerning nine children completed treatment for brain tumor was implemented. Identified problems in everyday life were linked to ICF codes. Descriptive statistics of ICF-linked code frequency supplemented by network visualization diagrams viewing the co-occurrence between codes within the body, activity participation, and environmental components were performed.

**Results:** Most documented problems were found in healthcare records, whereas the documentation in habilitation and school was sparse. The frequently occurring codes, independent of record source, were linked to the body function component, and ICF-linked problems in habilitation and school were salient in the activity and participation component. To gain a holistic picture of relations between ICF codes and problems, network visualization diagrams were used to illustrate clusters of problems.

**Conclusion:** Code prevalence likely reflects where healthcare professionals and educators focus their attention when meeting the needs of children treated for a brain tumor in context. To maximize the comprehensive view of functioning and participation of children in everyday life, the full range of difficulties regarding body impairments, activity limitations, and participation restrictions must be identified and linked to each other in patterns of co-occurrence, which the ICF facilitate. However, ICF provides no guidance on how to identify networks of problems within the body, activity, and participation. Identifying such networks is important for building comprehensive interventions for children.

## Introduction

Many children treated for brain tumors experience late effects that influence their ability to participate in everyday life (1). Diminished ability to take part in everyday life affects the health of the child, as human health is related to the ability to perform vital activities and participate in everyday life in supportive environments (2). In Sweden, brain tumor accounts for about one-third of childhood malignancies per annum, and many children with such diagnosis are expected to be long-term survivors (3). Despite being “cured” of their malignancy (4, 5), these children exhibit life-long problems in everyday life functioning. These problems can be related to cancer or its treatment and affect the psychosocial and cognitive abilities of a child (6–9). Compared to healthy peers and other cancer groups, children treated for brain tumors report poorer health-related quality of life (10, 11). Common symptoms include fatigue, which is often described as a distressing state (12), sleep disturbance, and headache (13). These children also experience scholastic difficulties with reading, writing, and doing math (14), impaired abilities to concentrate (15) and control their behavior (16, 17), and inattentiveness to social cues, which can lead to social exclusion by peers (18). School absence is more common among this group of children (19), and studies report that they have a poorer academic achievement compared to healthy peers (20–22).

For children returning to school after brain tumor treatment, informational exchanges between healthcare and school professionals are needed (8, 9). Professionals in healthcare, habilitation, and school (23, 24) are required by law to document the needs and support provided for a child. In healthcare and habilitation services, the planned and provided care, evaluations of the results, and recommended adaptations to improve outcomes are documented in the medical record of the child (23). In school, the developmental and learning needs of a child are documented in the individual education plan of the child. If a child is at risk of not achieving the overall educational goals, a written action plan with interventions and follow-up must be developed, with environmental adaptions included (25). The health and welfare team of the school also documents the difficulties and needs of the child (26). However, the documentation within healthcare almost exclusively focuses on medical aspects of the child's functioning (27), and the school documentation is fragmented. Moreover, linkages between problems described and actions planned are usually not as explicit in school documentation as within healthcare documentation (28). Providing comprehensive support to children treated for a brain tumor requires that users of the records not only read the information provided by their own services, but also link that information to the information provided by other services. Useful services require that the difficulties of the child are described and explained within the context in which the difficulties are being expressed and the support is to be provided (29, 30). A coherent plan for multilevel interventions to support children with brain tumors requires professionals across healthcare, habilitation, and school settings to understand how problems are transformed and linked when moving from body function to participation in school activities and other aspects of everyday life (31). Therefore, the co-occurrence of problems and the relationship between problems are just as important as single problems when providing services. Different services must identify strategies to enhance communication and collaboration to facilitate a comprehensive view of the child. By using the WHO's International Classification of Functioning, Disability and Health (ICF) (32), the understanding of the child's reality can be increased (33, 34) as the ICF goes beyond medical diseases presented in the International Classification of Disease (ICD-10) (35). The ICF uses a universal frame of language to holistically describe the functioning and health of the person with a possibility to use across disciplines and settings (33).

A common use for ICF is to analyze patterns in what information professionals tend to look for when assessing functioning. This is done by linking items from existing assessment instruments to ICF using established linking rules (36), even if the instruments not originally were developed based on the ICF. Further, Klang Ibragimova et al. (37) linked free texts from habilitation plans not originally based on ICF, to ICF codes as in the present study. In this study, the codes were applied to healthcare, habilitation, and school documents to describe what professionals within different services have focused on when documenting problems that children treated for brain tumors experience in their everyday life. Although the ICF manual (2001, 2021) provides guidance in how to code functioning into components and codes, the manual provides no guidance in how to analyze relations between components and codes within components, i.e., there is no guidance in the ICF manual about how to interpret and manage the bi-directional arrows in the ICF model that links the different components. To analyze co-occurrence and relations, a graphical model with a visualization network will be used in this study to view the connection between ICF as a roadmap to understand the associations between different aspects of human functioning, not provided by the current ICF classification system. Graphical models have been a useful tool in earlier studies with ICF to visually view the dependence structure of health aspects among individuals with a chronic health condition (38, 39).

### Aim

The study's aim was to use the ICF classification system to describe how professionals in healthcare, habilitation, and school document problems with everyday life functioning at body, activity, and participation levels for children who completed brain tumor treatment.

### Research Questions

How are the ICF codes identified in documents distributed within and across the services of healthcare, habilitation, and school?

How are the identified ICF codes distributed within the different ICF components: body function, body structure, activity/participation, and environmental factors?

How do the identified ICF codes co-occur and relate to each other within and between the components body, activity, participation, and environment?

## Materials and Methods

### Design

This study used a retrospective multi-method design.

### Approach

Overall, the study used a deductive content analysis approach (40) directed by the ICF. The ICF categorizes health information into four interacting components: body function (b), body structure (s), activity and participation (d), and environmental factors (e). The component body function (b) and body structure (s) comprise physiological and psychological functions of systems and anatomical construction of the body. The component activity and participation (d) comprise aspects of functioning from individual and societal perspectives. The activity and participation component includes two constructs: activity and participation. Activity describes the execution of a task or action of an individual, and participation describes the involvement of an individual in a life situation. The environmental factor component (e) addresses the various environments in which the individuals live and conduct their lives and the physical, social, cultural, and political features of those environments.

Each component of the classification can be categorized at four levels, ranging from ICF chapter (a letter accompanied by zero digits, indicating the lowest level of detail) to ICF code numbers with detailed definitions (a letter plus four digits indicating the highest level of detail). An example of this is within the component body function (b, which is the lowest or first level of detail), the first chapter describes *Mental functions* (b1, which is the second level). *Energy and drive functions* describe global mental functions (b130, which is the third level), and further the *Motivation Mental functions* describe the incentive to act; the conscious or unconscious driving force for action (b1301, which is the fourth or highest level of detail).

This study investigated the abilities of an individual to perform tasks or actions independently separately from their participation in life situations. The rationale is that individuals who lack the ability to perform tasks independently can nonetheless participate independently in life situations. The ICF manual (p.20 Swedish version, 2021) suggests four alternatives for ICF users who prefer to separate activity and participation when applying the activity/participation component of the ICF. The alternatives are: (a) define certain domains as activity and others as participation with no overlap, (b) as in alternative (a) and allowing overlap, (c) define all specified codes as activities and use first and second levels as participation, or (d) use both activity and participation codes in all domains. This suggestion is further elaborated in Appendix 3 of the ICF manual (41). Alternative (a) was applied in this study. As suggested in Appendix 3 of the ICF manual (41), the first four domains of the activity/participation component (e.g., learning and applying knowledge, general tasks and demands, communication, and mobility) were defined as activity and instead of “d” like in domain, these chapters were assigned an “a” like in activity. For the subsequent six domains (e.g., personal care, domestic life, interpersonal relationships, important life areas, and societal and civic life), p (participation) was used, rather than d (domain).

### Setting

Pediatric oncology departments serving urban and urban–rural areas in central Sweden provided access to medical records for children treated for brain tumors. Yearly these hospitals serve about 50 children (aged 0–18 years.) diagnosed with a primary brain tumor. The pediatric oncology departments provide surgical removal of the tumor to the extent possible, plus adjunct chemotherapy, and radiotherapy. Municipal hospitals deliver some types of chemotherapy, manage acute complications (e.g., neutropenia), and monitor children for persistent and late onset treatment-related complications (e.g., imagines). Child and youth habilitation focus on rehabilitation of the skills of the child. University hospitals link the children with municipal hospitals, habilitation services, and mainstream and/or special schools depending on the needs of children and where they reside. In total, two University hospitals, four municipality hospitals, and five habilitation services were involved in this study.

### Participants

Convenience sampling was used to recruit children of either sex and diverse ages with various types of brain tumors and other clinical characteristics. Children who received care at either of the two participating pediatric oncology departments were eligible if (a) they had been diagnosed with a brain tumor between the ages of 5 and 15 years and (b) completed a neuropsychological assessment about 1-year after ending treatment for their brain tumor.

### Procedure

Verbal and written information about the study was provided to eligible families by the consultant nurse for pediatric brain tumors at participating University hospitals. The nurse also asked for permission to share contact information for families interested in learning more about the study with the first author (A-CB), who telephoned the families and provided additional information about the study and answered their questions. After taking time to reflect on the requirements of the study, parents and children (age 15 years and older) signed the consent forms and mailed the documents and information about the schools of the child to the first author.

The heads of the involved hospitals and habilitation services facilitated the access of the author to the electronic healthcare records of the child, where documentation about the child by various healthcare professionals (e.g., nurses, physicians, and psychologists) was found. One hospital provided printed copies of the health records of the child. Participating children were linked to 19 schools during their education. Principals at these schools provided printed copies of documentation about the child by school professionals (e.g., teachers, special education teacher, and school-nurse).

### Data Extraction

The first author extracted data from the records of children between May 2018 and March 2019; each extraction was dated. The extracted data included the date of the documentation document source (service), the profession of the documenter, and the text that described problems regarding the function of children in everyday life. Data were extracted from the first neuropsychology assessment performed by the children around a year after the treatment completion. A problem was defined as a perceived difference between the present and desired state of functioning of the child (42) at the time of documentation. Time periods covered by the documents of interest ranged from 2.7 to 10.4 years (mean 5.1 years). In total, the extracted data comprised 847 pages and 182,014 words.

### Data Coding

The extracted text (units for analysis) was linked to ICF codes. The coding process was guided by a modified version of ICF-linking rules described in eight steps (36). The *first rule* highlights the need to require good knowledge of ICF fundaments. The first author was initially a novice user of the ICF classification system but became familiar with coding during the analysis through close mentorship and regular monitoring by two of the authors (MG and MB), who have extensive knowledge of the ICF and experience linking data to its codes. The *second rule* is that meaningful segments of text should be linked to the most precise, that is, the highest level of detail possible for relevant ICF codes. In this study, all documents were read through several times to become familiar with the content. If several problems appeared in an extracted text segment, that segment was divided into several meaning units with one problem per unit. The first author labeled each unit with word(s) relating to the problem and then linked the word(s) to the highest level of detail possible for the relevant ICF code. Problems related to body function/structure were linked to b1–b8 and s1–s8 codes. Problems related to activity in the activity/participation component were linked to Chapters a1–a4. Problems related to participation were linked to p5–p9. Lastly, the text related to environmental factors was linked to e1–e5. Table 1 shows an example of the coding process.

**Table 1:** Examples of the coding process.

**Meaning unit**	**Identified meaning of the problem**	**ICF chapter**	**ICF codes**
Otherwise have diffuse abdominal pain during the last week	Pain	b2 Sensory functions and pain	b280 sensation of pain
Difficult to undertake activities	Undertake activities	a2 General tasks and demands	a230 carrying out daily routine
Phone call with the mother: Child expressed school not being fun and having no friends	No friends	p7 Interpersonal interactions and relationships	p750 informal social relationships
Special support needed during lessons, does not work among all the teachers in school	Educational support	e3 Support and relationships	e330 People in position of authority

The *third rule* state that if a meaningful concept is not explicitly named in the ICF manual but can be related to the ICF code “other specified,” this should be avoided when possible. The *fourth rule* describes that when a lower level ICF code could not be found you should avoid using “unspecified categories” and instead use a higher level of ICF code. This was used when an explicit ICF code for the problem could not be found, but the chapter level was clear. The *fifth rule* states that when a meaningful concept cannot be linked to a specific ICF code of category, it was assigned as “not definable.” *Rules six* (assigning meaningful concepts to personal factors), *seven* (assigning meaningful concepts that cannot be found in ICF to not covered), and *eight* (assigning meaningful concepts referring to a diagnosis to a health condition) were not used, as personal factors or diagnosis were not the focus of the present study.

Problems related to the text segments with equivocal links or an ambiguous link to an ICF chapter or code were marked and discussed by the first author and the last (MB) author until they agreed on the most suitable ICF chapter and code. A second rater (MG), familiar with ICF and coding data to ICF, reviewed about 10% of the coding among a randomly selected data from all the participants. When discrepancies were identified, the data segments were discussed and agreements regarding the most relevant codes were made among the authors (A-CB, MG, and MB).

### Statistical Analysis

Frequencies of ICF codes were calculated based on occurrences of text segments with specific codes in the document. To generate information about relations between co-occurring codes for the body, activity/participation, and environmental components, network visualization diagrams of the frequency with which codes co-occurred together were created. First, all ICF codes were retrieved from the research records of participants. RIS files were created for each participant and time-period (year) since the first neuropsychological assessment after ending brain tumor treatment. Then, these data and VOS viewer software (43) were used to create a code co-occurrence network visualization, wherein each node represents an ICF code. The size of a node indicates the number of occurrences of the code; larger nodes indicate greater frequencies. Nodes located closer to each other have a stronger relatedness in terms of co-occurrence than those further apart and are visualized as more central in the diagram. The links indicate co-occurrence of two or more codes and the thickness of the link denotes link strength, in this case, the number of co-occurrences between codes. Sets of closely related nodes form networks, which are indicated by different colors. Unique abbreviated ICF code labels were added to the visualization (see **Figure 2** for the list of abbreviations and complete code labels). The network visualization diagram was visually inspected (based on content) to identify and name frequently occurring patterns of codes based on the ICF components body function and structure, activity, participation, and environment.

## Results

Parents of 12 children were approached about the study. Of these, 11 returned signed informed consent forms. Two children whose parents consented were not enrolled—one was diagnosed prior to age of 5 years and the other had yet to have a post-treatment completion neuropsychological assessment. Thus, the study sample consisted of nine children, including four females and five males with a mean age of 6.8 years (range 5–11 years). Five children lived in rural areas and four in urban areas. Children were diagnosed with medulloblastoma (5), astrocytoma (3), or craniopharyngioma (1). Six of them had received surgery + chemotherapy + radiotherapy, two surgery + chemotherapy, and one surgery + radiotherapy. Their grades in school at the time of diagnosis and data collection are presented in Table 2. Given the small sample size and risk to privacy, the details are sparse.

**Table 2:** Grade in school at diagnose and data collection.

**Grade**	**Number of children**
**Grade in school at diagnose**	
Pre-school	6
3rd grade	1
5th grade	2
**Grade in school at data collection**	
4th grade	2
5th grade	1
7th grade	1
8th grade	1
9th grade	2
1st yr. in high school	2

The overall result of reviewing records from nine included children, 4,543 problems linked to ICF codes representing both unique (mentioned once) and more common (mentioned repeatedly) problems were identified. The ICF codes were distributed among all, but one ICF Chapter: *s5 Structures related to the digestive, metabolic, and endocrine systems*. Within Chapters, 82 ICF codes were related to body function, 23 codes were related to body structure, 45 were related to the activity part, 22 codes were related to the participation part, and 26 codes were related to environmental factors. Some codes (154/4543) were only linked to chapter level as no code on the second level could be identified, and 69 codes were identified as not definable when being too diffuse to relate to any ICF code or to the study aim.

### Distribution of Identified ICF Codes Within and Across Services

**Figure 1:**
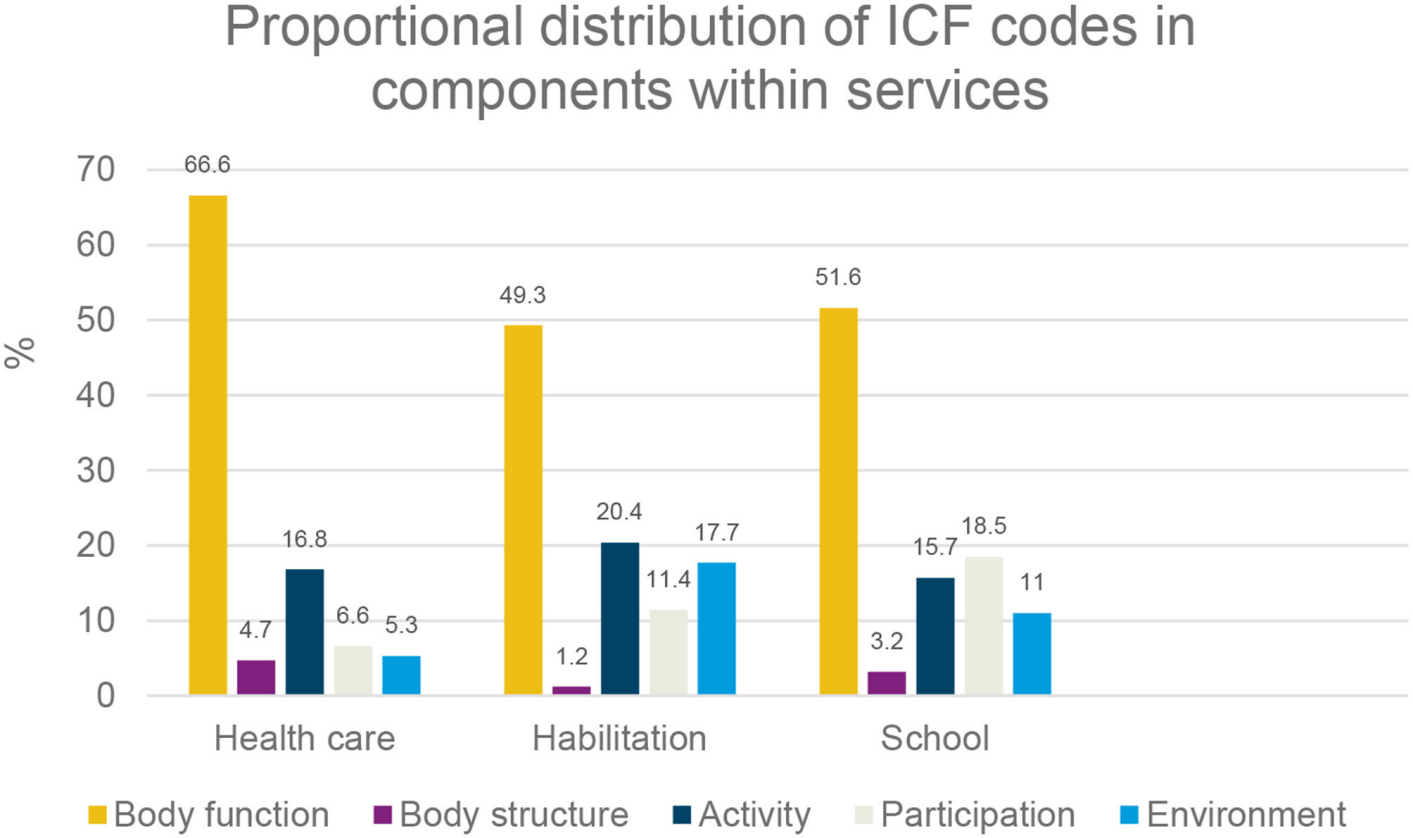
Distribution of codes in the ICF components within services.

Within each service type (healthcare, habilitation, and school) and overall, the largest proportion of ICF codes were linked to body function. Codes linked to body structure represented the smallest proportion of codes per service and overall. The proportion of codes linked to activity was the lowest, and the proportion linked to participation was the most prominent within school documentation. Habilitation accounted for the greatest proportion of codes linked to environmental factors, see Figure 1.

### Distribution of ICF Codes Within the Body Function, Body Structures, Activity/Participation, and Environmental Factors Components

The greatest proportion of problems linked to ICF codes was within the body function component (64%). Codes within the body structure component were sparse (4%). The proportion of codes within the activity component was modest (17%), as were codes within the participation part (8%) and in the environmental factors component (7%).

### ICF Codes Within the Body Function and Body Structure Component

The highest proportions of problems linked to ICF codes within the body function component (*n* = 2,882) were found in Chapter b1 Mental functions (48%) followed by the Chapters of b7 Neuromusculoskeletal and movement-related functions (22%) and b2 Sensory functions and pain (13%). A considerable proportion of ICF codes were in Chapter b5 Functions of the digestive, metabolic, and endocrine systems (10%). Few codes were in Chapter b3 Voice and speech functions, b6 Genitourinary and reproductive functions, or b8 Functions of the skin and related structures.

Within Chapter b1 Mental functions, ICF codes *b126 Temperament and personality functions* and *b130 Energy and drive functions* were present in 9/9 cases. In Chapter b2 Sensory functions and pain*, b280 Sensation of pain* accounted for almost 50% of the codes and was found in 8/9 cases. Within Chapter b7 Neuromusculoskeletal and movement-related functions, *b730 Muscle power functions* had about 30% of the codes and was present in 7/9 cases. An example of the most frequently used body function codes within each chapter are presented in Table 3.

**Table 3:** Examples of most frequently used ICF codes within the chapters of Body function component.

**ICF Chapter and number of codes within**	**ICF code**	**Number**	**Presented in number of cases**
b1	*b126 temperament and personality functions*	*n* = 103	9/9
Mental functions	*b130 energy and drive functions*	*n* = 380	9/9
	*b140 Attention functions*	*n* = 137	7/9
	*b144 memory function*	*n* = 177	8/9
	*b147 psychomotor functions*	*n* = 63	6/9
	*b152 emotional functions*	*n* = 63	7/9
	*b164 higher-level cognitive functions*	*n* = 93	7/9
b2	*b210 seeing functions*	*n* = 90	9/9
Sensory functions and pain	*b280 sensation of pain*	*n* = 188	8/9
b3	*b310 voice functions*	*n* = 23	2/9
Voice and speech functions			
b4	*b450 additional functions of the respiratory systems*	*n* = 29	4/9
Functions of the cardiovascular, hematological, immunological, and respiratory system	*b455 exercise tolerance functions*	*n* = 22	2/9
b5	*b535 sensation associated with the digestive system*	*n* = 58	8/9
Functions of the digestive, metabolic, and endocrine systems	*b555 endocrine gland function*	*n* = 43	8/9
b6	*b610 urinary excretory functions*	*n* = 14	3/9
Structures related to the genitourinary and reproductive systems	*b620 urination functions*	*n* = 16	2/9
b7	*b730 muscle power functions*	*n* = 188	7/9
Neuromusculoskeletal and	*b735 muscle tone functions*	*n* = 79	5/9
movement-related functions	*b755 involuntary movement reaction functions*	*n* = 128	9/9
	*b760 control of voluntary movement functions*	*n* = 73	8/9
b8	*b810 protective function of the skin*	*n* = 11	4/9
Skin and related structures			

The body structure component had the lowest number of identified problems linked to ICF codes (*n* = 193). The largest proportion of ICF codes in this component were found in the Chapter s1 Structures of the nervous system (65%), followed by s7 Structures related to movement (17%) and s8 Skin and related structures (9%).

### ICF Codes Within the Activity Part of Activity/Participation

The highest proportion of problems linked to codes within the activity component (Chapters a1 to a4; *n* = 776) was found in Chapter a2 General tasks and demands (41%) followed by a1 Learning and applying knowledge (31%). The proportion of ICF codes in Chapter a4 Mobility was moderate (21%) and the lowest proportion of ICF codes in Chapter a3 Communication (7%). Within Chapter a1 Learning and applying knowledge accounted for about 45% of the codes in *a166 Reading* and *a170 Writing*. The codes *a210 Undertaking a single task* and *a230 carrying out daily routin*e displayed most of the identified codes within Chapter a2 General tasks and demands and were present in 8/9 cases. In Chapter a4 Mobility, the codes *a440 Fine hand use* (36%) and *a455 Moving around* (24%) accounted for the greatest proportions of codes.

### ICF Codes Within the Participation Part of the Activity/Participation

The highest proportion of problems related to ICF codes within the participation component (*n* = 370) were found in Chapter p7 Interpersonal interactions and relationships (46%), followed by the Chapter p8 Major life areas (35%). ICF codes found in Chapter p5 Self-care accounted for 16% of the codes in participation, and the code *p550 Eating* was present in seven of nine cases. The ICF code *p820 School education* was the most frequently documented code (32%) and seen in eight of nine cases.

### ICF Codes Within the Environmental Factors Component

The largest proportion of problems linked to the ICF codes within environmental factors (*n* = 322) was found in Chapter e3 Support and relationships (45%), followed by Chapters e1 Products and technology (17%), e2 Natural environment and human made changes to the environment (14%), e4 Attitudes (14%), and e5 Services, systems, and policies (10%). In Chapter e2 one ICF code related to “other specified” (e298 Natural environment and human-made changes to environment) despite recommendations not to use other specified occurred 15 times. The ICF code *e310 Immediate family* was the most frequently occurring code within environmental factors (21%) and was present in all cases. The ICF code *e330 People in position of authority* was also frequently documented (18%) and was present in seven of nine cases.

### The Relation of ICF Codes Within and Between ICF Components

**Figure 2:**
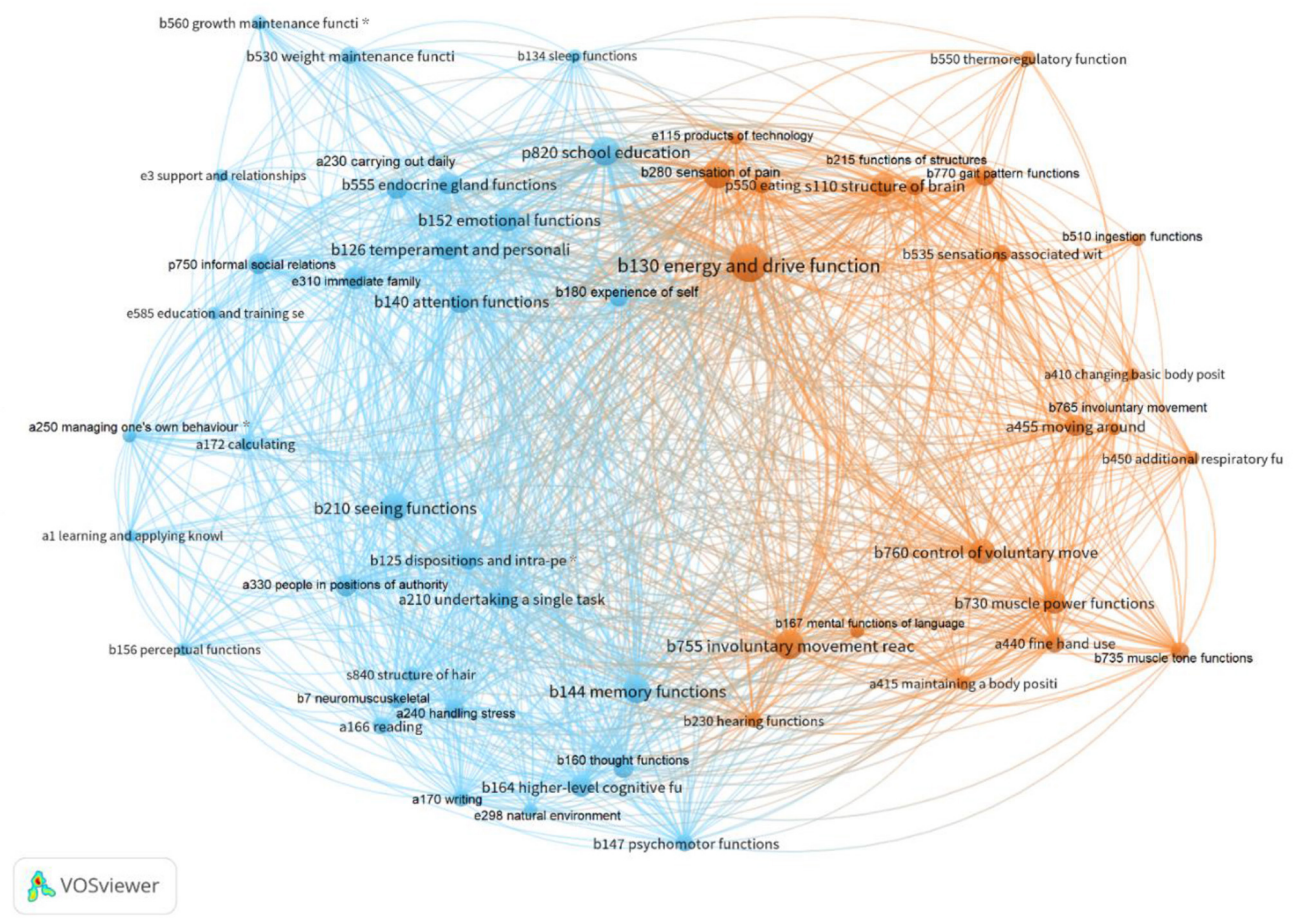
Network visualization diagram within and between the ICF components. ICF- codes with labels (shortened), co-occurrence threshold = least 10 occurrences, No. of codes = 54, No. of clusters = 2. Blue cluster codes; b134 sleep functions, b125 dispositions and intra-personal functions*, b126 temperament and personality functions, b140 attention functions, b144 memory functions, b152 emotional functions, b180 experience of self and time functions, b210 seeing functions, b530 weight maintenance functions, b560 growth maintenance functions*, b730 muscle power functions, b750 motor reflex functions, b760 control of voluntary movement functions, a166 reading, a170 writing, a172 calculating, a210 undertaking a single task, a240 handling stress and other psychological demands, a250 managing one's own behavior*, a330 Speaking, a350 conversation, p750 informal social relationships, p820 school education, e250 sound, e298 natural environment and human-made changes to environment, other specified, e310 immediate family, e330 people in positions of authority, e585 education and training services, systems, and policies. Orange cluster codes; b130 energy and drive functions, b230 hearing functions, b280 sensation of pain, b510 ingestion functions, b535 sensations associated with the digestive system, b550 thermoregulatory functions, b730 muscle power functions, b735 muscle tone functions, b755 involuntary movement reaction functions, b760 control of voluntary movement functions, b770 gait pattern functions, a410 changing basic body position, a415 maintaining a body position, a440 fine hand use, a455 moving around, e115 products and technology for personal use in daily living. *ICF codes included in the old version of ICF-CY.

**Figure 3:**
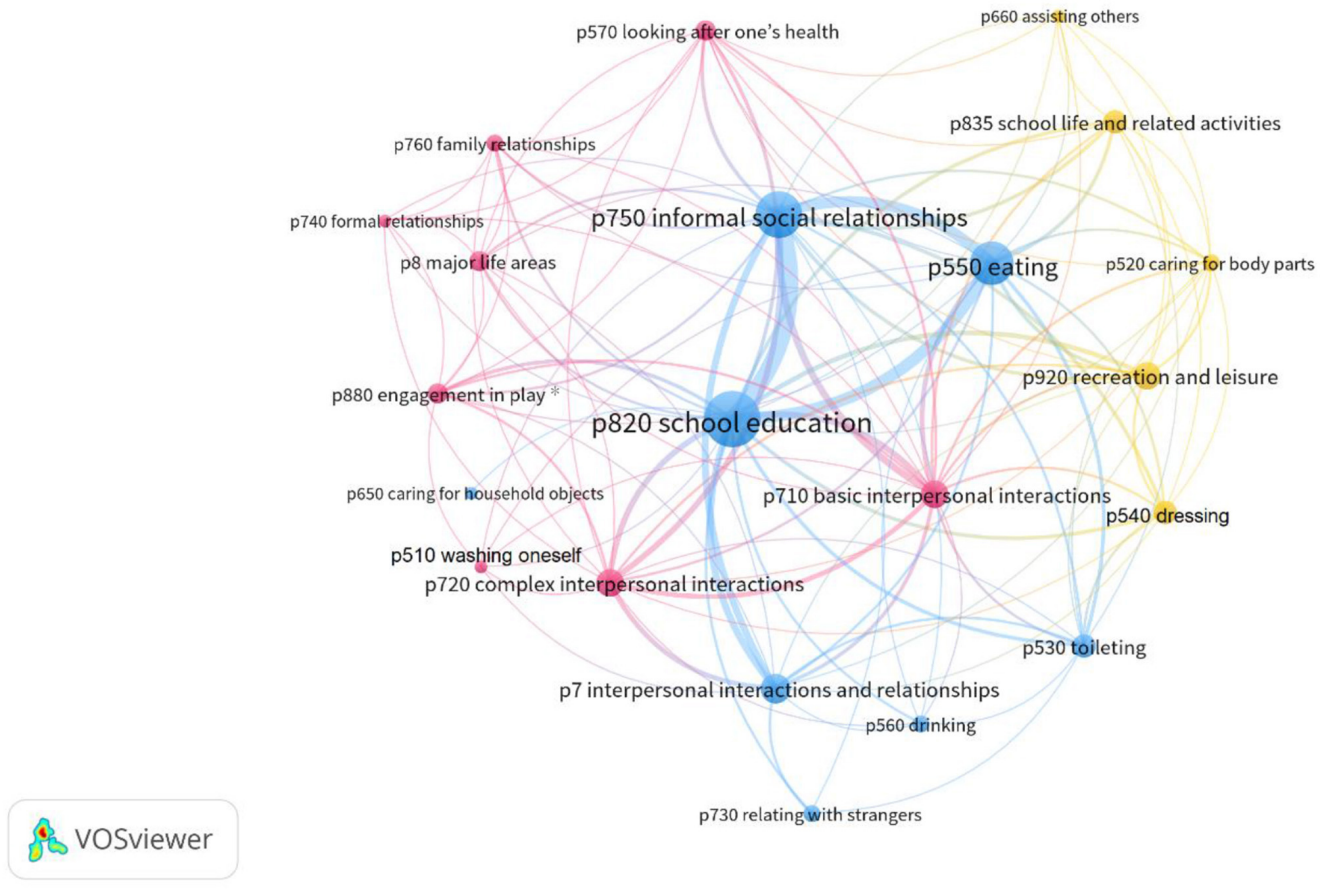
Network visualization diagram between the participation codes. ICF p codes with labels, co-occurrence threshold = least 1 occurrence, No. of codes = 21, No. of clusters = 3. *ICF codes included in the old version of ICF-CY.

The RIS files created for building network visualization diagrams resulted in 193 unique ICF codes and since proportionally fewer (p) participation codes occurred more than 10 times, a network visualization diagram based on all p-codes and their linkages to b-, a-, and e-codes was created (**Figure 3**). The final network visualization diagram (Figure 2) is based on the codes that occurred at least 10 times. The diagram displays two clusters of networks: one of these clusters is comprised of blue nodes and links, and the other by orange nodes and links. The color signifies nodes and links that tend to co-occur, that is the clusters of problems that, as indicated by the thickness of links, likely share an underlying cause. Nodes in the center of the diagram tend to relate to both clusters as both blue and orange links to these nodes. Central nodes tend to be large since the codes occur frequently and have many links to both the blue and orange cluster. Here, the central nodes seem to be the body function codes *b130 energy and drive functions, b144 memory functions, b180 experience of self and time functions, b280 sensations of pain*, and *b755 involuntary movement reaction functions;* the Participation code *p820 school education*, and the environmental factors code *e330 people in position of authority*. Note that no activity (a) code is central. A common theme of the central nodes seems to be information processing.

The nodes in the blue cluster (see Figure 2) can be characterized as related to cognition and managing school tasks. The network shows that problems in body function related to sleep, seeing, weight, and growth maintenance (*b134, b210, b530, b560*) and personality functions (*b125, b126, b152*) tend to co-occur with problems with attention, memory, and experience of self and time (*b140, b144, b180*) and to problems in basic learning and reading, writing, and calculating (*a166, a170, a172*). Problems related to sleep and seeing also link to carrying out tasks in school (*a210*), managing stress (*a240*), and managing behavior (*a250*). In addition, problems in speaking, conversation, social relationships (*p750*) and education (*p820*) is in this network. The orange cluster also includes problems concerning support and relationships (*e3*) within and outside school.

The cluster of orange nodes in the network can be characterized as illustrating the co-occurrence of problems related to using movements in everyday life and regulating sensations such as hunger, temperature, and pain. A central problem is energy level (*b130*). Problems related to using movements in everyday life and regulating sensations tend to co-occur with problems related to pain and hearing (*b230, b280*), problems with movement related functions (*b730, b735, b755, b760, b770*), problems with mobility functions (*a410, a415, a440, a455*), and problems with metabolic functions (*b510, b535, b550*). Needing equipment/assistive technology in daily life (*e115*) seems to be linked to the orange network.

Few participation problems were documented in the records; thus, these problems do not appear as nodes in a network visualization based on at least 10 occurrences of a code. Therefore, another network visualization (see Figure 3) was created based on the participation (p) codes that occurred at least once. Three networks of co-occurring problems were identified: blue, pink, and yellow. Again, nodes with a central position are more prominent, tend to be linked to all or several of the identified networks, and perhaps greatly influence the participation of children in everyday life. Central participation (p) nodes seem to be participation in basic interpersonal interactions, informal social relationships, eating, and school education. The common theme is social interaction and relationships. See Figure 3.

As seen in Figure 3, the blue cluster in the network contains four relatively larger nodes (informal social relationships, interpersonal interactions and relationships, school education, and eating); the common theme seems to be informal relationships outside family/home. Three of these nodes (informal social relationships, school education, eating) frequently co-occur and thus have strong links to each other. The pink cluster seen in Figure 3 contains eight nodes whereof two are larger, basic interpersonal interactions and complex interpersonal interactions; these nodes also have frequent links to the central node interpersonal interactions in the blue cluster. This pink cluster contains nodes for formal relationships, family relationships, engagement in play, and looking after one's health; the common theme seems to be problems with relationships within the family. A common theme of the pink cluster of nodes in Figure 3 seems to be participation in formal/organized activities. The yellow cluster in the network in Figure 3 contains five nodes that are less central in the diagram, indicating lower strength of their relatedness to the overall network. School life and related activities and recreation and leisure are relatively large nodes that also are linked to the pink network. The nodes representing caring for body parts and dressing are smaller.

## Discussion

Long term effects of brain tumor treatment concern not only body function but also the performance of activities and participation in everyday life activities. Therefore, problems on all these levels must be analyzed as patterns of problems based on relatedness and co-occurrences to obtain a comprehensive view. This study reveals that, irrespective of service type, professionals involved in services for children who completed brain tumor treatment focus their documentation regarding problems of the child on body function aspects (physical and psychological).

Linkages between body function focused problems and performance of everyday life activities are somehow invisible in the records. This invisibility is because co-occurrences are not explicitly documented, which obstruct the development of a comprehensive bio-psycho-social understanding of the problems that children experience following treatment for a brain tumor. To address the co-occurrence of problems, we linked information in the various service records to ICF codes and used the coding to generate network visualizations. The results confirm the ICF classification system as a useful tool for coding information in records to inform a more comprehensive understanding of children's problems and their need for support following treatment for brain tumor. ICF manual in its current form still do not provide users with guidance on how body, activity, and participation codes co-occur and are related to each other. Although the previous child and youth version of ICF (ICF-CY) now is completely merged into the ICF classification, this study started out by using the ICF-CY for identifying codes for analysis. Noten et al. (44) noted that the ICF in its current form does not cover child-specific codes to the same extent as ICF-CY. The codes identified were therefore in a *post-hoc* analysis compared to ICF (2021). The comparison revealed that four codes identified in ICF-CY (b125 disposition and intrapersonal functions, b560 growth maintenance functions, a250 managing one's own behavior, and p880 engagement in play) were not found in the new merged version of ICF. Three of these codes occurred so frequently (more than 10 times) that they appear in the network diagrams (b125, b560, a250). Therefore, in the analysis, the four ICF-CY codes were kept although not seen in the merged ICF version. This approach illuminated relations and co-occurrences between body function, activity, and participation codes. The visualizations provide some information, but still additional contextualization of what codes stands for within a certain population is needed to obtain a comprehensive view of everyday function.

Findings from this study reveal that most of the documented problems (80%) were found in healthcare records, which may not surprise, as healthcare bears the main responsibility for the medical follow-up of the child after ending brain tumor treatment. On the other hand, habilitation, and school play critical roles in assisting the children to maximize their functioning in everyday life and their quality of life. Long-term follow-up of children treated for brain tumors and other malignant conditions is guided by medical protocols and evidence-based recommendations (45), where the main focus of which are medical outcomes (e.g., endocrine function, cardiac function, linear growth, and weight category). Guidelines regarding comprehensive assessment and, moreover, intervening to support the functioning of the child in everyday life are lacking. Children typically meet a physician and a neuropsychologist at certain timepoints after ending cancer-directed treatment. Depending on the treatment exposures and identified healthcare needs of the child, a multiprofessional assessment is done, and a rehabilitation plan with interventions and goals is generated with regular re-assessments and revision of the plan as indicated (45). Guidelines in congruent long-term follow-up remain a vision and are not yet fully implemented at childhood cancer centers throughout Sweden.

Habilitation contributed 11% of the documented problems. Of note is that two of nine children in the study did not have access to the habilitation service, which partly explains the low proportion of problems documented by habilitation. However, 9% of the documented problems were derived from school records, and all of the children in the study attended school, which is mandatory (26). The limited documentation by school personnel could be due to the use of standardized educational plans where documentation of regular evaluations and adjustments are not mandatory (46). Previous studies have found that school professionals avoid documenting psychosocial issues to protect the personal integrity of children and their guardians (30). Thus, this information tends to be exchanged orally between colleagues (47).

Problems documented in habilitation and school were mostly linked to body function codes, and terms used by healthcare were adopted by professionals providing habilitation and educational services. This tendency is problematic; in that, the support provided in habilitation should be based on the needs of children for support in everyday life functioning, which is not the focus of the healthcare professionals (48). Schools focus on promoting and supporting the educational and social development of the child to reach the educational goals and become independent individuals (24). The multidimensional approach that should be seen in the school context was partly confirmed by the ICF codes linked to activity, participation, and environment, even if they were sparse. Yet, problems linked to body function codes dominated in school documents. To support a comprehensive approach to understanding the needs of children and developing interventions to maximize their overall health and daily functioning, the ICF classification may help professionals across services to broaden their perspectives on the health of the child and collaborate to assure that children are offered the comprehensive array of services they need (49).

Problems linked to codes from the chapter of mental functions (b1) in the body function component were frequently mentioned and problems linked to the ICF code *energy and drive functions (b130)* were noted for each participant. Prior studies have identified “cancer-related fatigue” as a common problem for children who completed brain tumor treatment and the association between fatigue and problems performing daily activities (12, 50, 51). Studies have also concluded that fatigue is associated with problems regarding aspects of cognitive functioning, such as slow processing speed, mental health problems, such as depressive symptoms (52, 53), and problems with social interactions (53). Within the chapter on mental functions, other ICF codes to which problems were linked concerned personality functions. Personality functions have been highlighted earlier in research regarding these children described having a low self-esteem, worse mood, and lower level of stress tolerance (50).

Even though they had completed treatment, most of the children (8/9) had documented problems linked to *pain* (*b280*). This finding is consistent with prior studies, which found that pain from headache is distressing for children post-treatment for brain tumors (50, 54, 55). Problems linked to motor functions (b7) presented in all nine children: poor muscle tone, balance disturbances, and poor coordination of movements occurred frequently. These motor function problems are highlighted in previous studies (56, 57), as causing limitations in everyday life activities for brain tumor-treated children. Motor function problems affect the ability of children to play with friends during school breaks and leisure time activities. The current study identified documented problems with *informal social relationships (p750*), which includes socializing with friends. This is consistent with prior studies, which found that children treated for brain tumors have problems in making and maintaining friendships and lack the capacity to engage in social activities in everyday life (50).

Most of the children (8/9) had problems linked to *school education (p820*), including attending regularly, organizing, and learning subjects within the educational program and reaching curriculum goals, which prior studies also found among cancer-treated children (58, 59). The present study also identified documentation of problems with specific aspects of learning and applying knowledge (a1) and with general tasks and demands (a2). This implies that the children had problems learning to read, write, calculate, and perform tasks, which is also highlighted in previous studies (60) and tend the treated children to perform poorly academically (50) and need special education support to a greater extent than other children (61).

Problems linked to environmental codes were rare. This lack may be explained by the framing of environmental problems as explanations for problems with the functioning of the child. Identified environmental problems tended to relate to the *immediate family (e310*). The family is the primary source of support for children; thus, the unit and each of its members are affected by the diagnosis of the child in various ways across the cancer trajectory (62). Parents may be overprotective, feel sympathy for the child, and experience difficulties with discipline and setting limits and consistent expectations for the behavior of the child (63). Children must receive needed support to maximize their adaptation and thus the ability to function in their environments, including within school. Professionals representing various services must collaborate around the child and their family. They need knowledge and skills about relating assessments of the problems of children to body functions, activity performance, and specific everyday challenges with functioning in the school and/or family environments. Such knowledge and skills in how to link different types of information are necessary in order to utilize the available environmental support with the aim to enhance functioning in everyday life activities (64).

The environmental problems identified by professionals also tended to be documented without a clear description of co-occurrences and relations between problems and primarily relate to body functions. This situation is problematic problems concerning functioning in an everyday context require developing and implementing environmental adaptations targeted toward problems on the body and activity levels. Such environmental adaptations are probably the main focus in participation interventions (65).

A comprehensive view of problems of children and needs for support to maximize functioning can be explicated in networks based on frequency, links based on co-occurrence, and centrality. Identifying such networks might be one solution toward unraveling the content of arrows between ICF components as shown in Figures 2, 3. Here, the network analyses are attempts to illustrate that problems tend to occur in networks, with some types of problems co-occurring more frequently and having stronger links to each other. In addition, some problems are more central in a network (more links to other nodes, that is, codes for problems), and some are more peripheral (fewer links).

Central body level problems seem to be *energy and drive functions (b130), memory functions (b144), experience of self and time (b180), sensations of pain (b280)*, and *involuntary movements (b755)*. Central problems related to participation were *informal social relationships (p750)* outside home*, family relationships (p760), basic interpersonal interactions (p710), and school education (p820)*. These identified problems are in line with what prior studies have identified as common problems seen in children treated for brain tumor (19, 63, 66, 67). However, the prior studies did not explicitly relate these common problems to each other. In the current study, these central problems seem to be related to the ICF codes for most of the other documented problems. The central position of these problems (nodes) also indicates that they are important for the two identified networks of problems where the blue network concern cognition (body level) and managing school tasks (activity level), and the other identified orange network seems to be related to moving around in everyday life (activity level) and metabolic function (body level). In clinical practice, professionals need to identify the networks of the problem of the child or an influential (dominant) cluster within the network as targets for interventions to support the child and maximize functioning in everyday life. Based on prior professional experiences and the results of prior studies (68–70), the collaborative problem solving (CPS) model might be one way to support the identification of the network of problems of an individual child and potential targets for interventions that address the multiplex of problems comprising a network or an influential cluster within that network. An initial step in applying the CPS model is to identify several problems and then potential explanations for those problems (71). For example, healthcare professionals might not readily link problems of a child with body function detected in healthcare to problems with performing activities in habilitation or to activity performance or participation in school or family life. Rather what is explicitly documented as a problem within one service can be seen as an explanation to a problem within another service. The differences in how problems and goals may be documented in healthcare, habilitation, and school services are illustrated in Table 4 in relation to the CPS model, containing the steps problem, explanation, goal, and method (42).

**Table 4:** Problems identified as targets for intervention by organizations serving the child.

**Problem**	**Explanation**	**Goal**	**Method**
*Pediatric oncology* Fatigue (energy level b130) as a treatment-related complication	Brain damage, long lasting side effect that may disappear with time	Not tired	Medication
*Habilitation* Seldom physical active, difficulties with staying on task (a210)	Acquired brain injury caused by brain tumor and treatment	Perform more physical activities, less tired	Training program, medication, and psychosocial support
*School* Seldom attend lessons, (p820), difficulty to finish school tasks (a230), do not reach curriculum goals (p820). Difficulty performing mathematical operations (a172)	Tiredness as side effect of treatment School activities not adapted to length and time points of alertness	Finish school tasks, reach curriculum goals	Setup opportunities for activities requiring less motor activity in breaks Adapt schedule, length of tasks and instruction to level of alertness

In conclusion, ICF codes identified from documentation in the records of the child, mainly focused on the problems related to body function aspects. For a comprehensive view of the functioning and participation of the child in everyday life, documentation should focus on problems related to activity limitation and participation restrictions, and their relations to body function and environmental characteristics. However, ICF does not provide guidelines for how to assess how ICF codes within and between its components relate to each other. Therefore, to support the functioning and participation of the child in everyday life, networks of problems related to body function, activity, participation, and environment must be identified. One way of doing this might be to use the CPS model.

### Limitations

This study focused on clusters of ICF-linked problems in everyday life, documented in records from healthcare, habilitation, and school for children after ending brain tumor treatment. The frequency of occurrence of ICF codes has been measured without qualifiers (from no to complete problem), that could have provided information on the severity of the problems. Using qualifiers is also recommended but was not possible due to the type of documents analyzed. To use written texts in the records means that some degree of interpretation has been done at the timepoint of professionals' documentation and may not reflect the everyday life context of the child (37). Frequencies of problems were calculated but not their impact on the everyday life of the child beyond the service settings. A weakness is the lack of two coders to establish the extent to which the identification of documented problems and linkages to ICF codes are reliable. That is replicable across reviewers and additional coders for some portion of the records is a means to enhance the reliability of the data extraction and coding process. Strategies to enhance the reliability of these processes and the validity of the results in this study included continuous dialog within the research group (MB and MG) where the senior researcher (MG) has expertise in applying the ICF. The author (A-CB) has expertise in the healthcare of children treated for brain tumor, and collaboration with habilitation and school. Alternatively, their prior research and clinical experiences may have unintentionally biased the results.

The strength of a co-occurrence network visualization is the graphical presentation of information about relatedness of codes (38, 44). Nonetheless, there is still a loss of information about the context in which the codes co-occur. In addition, depending on technical choices and the established cut-off set for the number of nodes in the visualization, the relatedness of nodes is not reflected with perfect accuracy in its two-dimensional presentation. Therefore, the conclusions that can be drawn from the visualization are limited and may not be upheld with different cut-points or larger sample sizes. Consequently, network visualizations are best used to generate hypotheses or provide additional support for expert judgments (72). This study represents feasibility testing of the use of network visualizations to identify clinical patterns that are important for planning comprehensive support to address the full array of problems with functioning in everyday life experienced by children treated for brain tumors.

### Clinical Implications

The results from this study provide an opportunity for professionals in healthcare, habilitation, and school to reflect on what domains require attention when meeting the child in context. Most children who survive treatment for a brain tumor will go on to require life-long healthcare monitoring for co-morbid medical conditions attributable to their treatment exposures and to experience problems with functioning in everyday life. Healthcare follow-up concerns evidence-based screening for body problems. Relatively little attention is given to the activity and participation of the child in everyday life. The environments where the child operates also matter and should be assessed. The results of the extraction and analysis of documentation by services supporting children treated for brain tumor highlight that these services differ in their goals for the child, which may not be communicated within and among the services to provide a comprehensive view of problems of the child to address via collaborations. Implications also include that the use of the ICF classification system is feasible in interdisciplinary settings, even though its application is complex and time-consuming for those who lack familiarity. The patterns of relationships between ICF codes presented in the network visualization diagram could enhance understanding the problem complexity in the heterogeneous group of children treated for brain tumors.

## Data Availability Statement

The data sets presented in this article include personally identifying information that present risks to confidentiality and cannot be shared without the expressed consent of the children/their parents. Therefore, we do not include an email address where requests for the data sets can be sent.

## Ethics Statement

This study was given formal approval by the Ethical Board in Linköping, Sweden (Dnr 2017/475-31). The participation in the study was voluntary, and parents and children were assured confidentiality. Given the small sample size and risk to privacy, the details about the study sample are sparse. The participants were also informed that they could withdraw from the study at any time without impact on the ordinary care or education of the child.

## Author Contributions

A-CB, MG, and MB involved in conceptualizing and finalizing the manuscript. SS involved in analyzing and drafting the manuscript and in language review. KE involved in conceptualizing the study and partly involved in the coding process. SC involved in analyzing the data mainly network visualization diagrams and drafting the manuscript. All authors contributed to the article and approved the submitted version.

## Funding

The Swedish Childhood Cancer Foundation (Barncancerfonden; Grant No. TJ2016/0032) supported funding for data collection and analysis. Jönköping University paid the publication fee.

## Conflict of Interest

The authors declare that the research was conducted in the absence of any commercial or financial relationships that could be construed as a potential conflict of interest.

## Publisher's Note

All claims expressed in this article are solely those of the authors and do not necessarily represent those of their affiliated organizations, or those of the publisher, the editors and the reviewers. Any product that may be evaluated in this article, or claim that may be made by its manufacturer, is not guaranteed or endorsed by the publisher.
